# Expression of Bim, Noxa, and Puma in non-small cell lung cancer

**DOI:** 10.1186/1471-2407-12-286

**Published:** 2012-07-12

**Authors:** Jun Sakakibara-Konishi, Satoshi Oizumi, Junko Kikuchi, Eiki Kikuchi, Hidenori Mizugaki, Ichiro Kinoshita, Hirotoshi Dosaka-Akita, Masaharu Nishimura

**Affiliations:** 1 First Department of Medicine, Hokkaido University School of Medicine, North 15, West 7, Kita-ku, Sapporo, 060-8638, Japan; 2 Department of Medical Oncology, Hokkaido University Graduate School of Medicine, Sapporo, Japan

## Abstract

**Background:**

The BH3-only members of the Bcl-2 protein family have been proposed to play a key role in the control of apoptosis and in the initiation of the apoptotic pathways. In this study, we evaluated the expression of Bim, Noxa, and Puma in non-small cell lung cancer (NSCLC).

**Methods:**

A total of 135 surgically resected NSCLCs were immunohistochemically assessed for Bim, Noxa, and Puma expression. The immunoscores were determined, and then its correlation with either the clinicopathological variables or the survival outcomes were analyzed.

**Results:**

Immunohistochemical reactivity for Bim, Noxa, and Puma was detected in the cytoplasm of the tumor cells. Bim expression was associated with several clinicopathological factors, including sex (p < 0.001), smoking habit (p = 0.03), pathological histology (p = 0.001), pathological T stage (p = 0.03), pathological disease stage (p = 0.02), and differentiation of tumor (p < 0.001). Multivariate logistic regression analysis showed a significant correlation between low Bim expression and squamous cell carcinoma (p = 0.04), in addition to a correlation between high Bim expression and well differentiated tumors (p = 0.02). Analysis of cellular biological expression demonstrated a link between low Bim expression and high Ki67. While Noxa expression was also shown to be correlated with both smoking habit (p = 0.02) and the pathological histology (p = 0.03), there was no strong association observed between the expression and the clinical features when they were examined by a multivariate logistic regression analysis. No correlations were noted between Puma expression and any of the variables. Our analyses also indicated that the expression levels of the BH3-only proteins were not pertinent to the survival outcome.

**Conclusions:**

The current analyses demonstrated that Bim expression in the NSCLCs was associated with both squamous cell carcinoma histology and tumor proliferation.

## Introduction

Lung cancer is the most common cause of malignancy-related death in the world, and despite advances in both detection and treatment, its incidence rate is still increasing. Although the development of several new agents and molecular targeted therapies has led to an increased survival of patients with non-small cell lung cancer (NSCLC), the actual cure rates remain low for advanced NSCLC patients [1,2]. In order to improve the poor prognosis of patients with NSCLC, further studies that specifically examine the clinical features of lung cancer in addition to the development of new therapeutic strategies will be required.

Deregulation of apoptosis has been demonstrated to lead to cancer development, proliferation, and treatment resistance [3,4]. The mitochondria-mediated apoptotic pathway is largely regulated by the Bcl-2 family proteins [5], with members of this family possessing at least one of four conserved motifs that are known as the Bcl-2 homology domains (BH1 to BH4). These domains have been divided into three subfamilies, with the BH3-only proteins, such as Bik, Bid, Bim, Bmf, Hrk, Bad, Noxa, and Puma, exhibiting sequence homology only in BH3 [5-7]. Bim is induced by the withdrawal of growth factors via either of the two major epidermal growth factor receptor (EGFR)-dependent pathways: the Raf/MAPK or the Akt/PI3K [8,9]. Noxa and Puma are transcriptional targets of p53 and have been shown to play an active role in p53-induced apoptosis [10,11]. Recent studies have also demonstrated that aberrations of the BH3-only proteins are linked to tumorigenesis in several cancers [12,13]. Furthermore, there has been an accumulation of evidence showing an association between the frequent loss of BH3-only proteins and a poor cancer prognosis [14-16]. Although previous data have demonstrated the expression of Bcl-2 family proteins in lung cancer [17,18], there has been very little information available on the actual role that these proteins might play in lung cancer, and thus, further studies are warranted.

In the current study, we used immunohistochemistry to examine the expression of Bim, Noxa and Puma in a series of NSCLCs. Subsequently, we attempted to determine if there were any correlations between the expression of these proteins and features such as clinical and clinicopathological parameters, cell biological characteristics, or survival outcomes.

## Materials and Methods

### Patient and Specimens

This study was approved by the Medical Ethics Committee of Hokkaido University School of Medicine. A total of 135 patients (91 males and 44 females) who underwent radical surgery between 1982 and 1994 at Hokkaido University Medical Hospital were included in the study. Informed consent was obtained from all patients prior to enrollment in the study. Histological diagnoses and grades of differentiation of the primary tumor specimens were determined in accordance with the 1982 World Health Organization criteria. These histological analyses demonstrated that 74 samples were adenocarcinoma (Ad), 54 were squamous cell carcinoma (Sq), 5 were adenosquamous cell carcinoma (AS), and 2 were large cell carcinoma (La). For the statistical analyses, tumor specimens were divided into either a Sq or non-Sq group, which included the Ad, AS, and La. The pathological stage (pStage) was based on the guidelines of the American Joint Committee on Cancer for post-operative tumor-lymph node-metastasis (TNM) classification [19].

### Immunohistochemistry

Immunohistochemical analyses were performed for Bim, Noxa, Puma, or Bcl-2 using 4 μm-thick histological sections that were cut from formalin-fixed, paraffin-embedded blocks. After the sections were deparaffinized in xylene and then rehydrated in ethanol, the sections were treated in 10 mM citrate buffer, pH 6.0 for 20 minutes at 121°C in an autoclave in order to retrieve the antigenicity. To block the endogenous peroxidase activity, the sections were subsequently immersed in methanol containing 1.5 % hydrogen peroxide for 20 minutes followed by incubation with normal rabbit serum in order to block the nonspecific antibody binding sites. Sections were incubated at 4°C overnight with either anti-Bim/Bod rabbit polyclonal antibody (Sigma-Aldrich corp. St Louis, MO, USA), diluted 1:300, primary anti-Noxa rabbit polyclonal antibody (Biovision, Inc., Mountain View, CA, USA), diluted 1:200, primary anti-Puma rabbit polyclonal antibody (ab27669, Abcam, Cambridge, MA, USA), diluted 1:50, or primary anti-Bcl-2 mouse monoclonal antibody (clone124, DAKO, Tokyo, JAPAN), diluted 1:40. Immunostaining was performed by using the biotin-streptavidin immunoperoxidase method with 3,3-diaminobenzidine as the chromogen (SAB-PO kit; Nichirei, Tokyo, Japan). Hematoxylin solution was used for counterstaining. For each batch of sample that was stained, one sample that was positive for either Bim, Noxa, Puma, or Bcl-2 was included as a positive external control. Using a BX 40 microscope (Olympus, Tokyo, Japan), the immunohistochemical evaluations were assessed twice by a single investigator (J.S.K.) who was blinded to the status of the other immunohistological and clinical data.

### Immunohistochemical Scoring

Bim, Noxa, and Puma staining levels were classified as either high or low, as per a previously described method [17,18]. To be defined as having a high expression of Bim, Noxa or Puma, moderate-to-strong staining intensity had to be observed in more than 50 % of the tumor cells. Samples were scored as low when the expression was present in less than 50 % of the tumor cells. Immunohistochemically staining sections were judged high expression for Bcl-2 expression when more than 10 % of cancer cells showed cytoplasmic staining [20,21].

### Western Blot Analysis

Cell lysates of Jurkat cells, the NSCLC cell line (HCC2429), and tumor tissues were prepared by lysing cells and tissues in radioimmune precipitation assay buffer (150 mM NaCl; 1 % Triton X-100; 1 % B deoxycholate; 0.1 % sodium dodecyl sulfate (SDS); 10 mM Tris, pH 7.4) supplemented with 100 μg/mL leupeptin, 100 μg/mL aprotinin, and 10 mM phenylmethylsulfonyl fluoride. Protein samples were resolved using SDS-PAGE gels, transferred onto nitrocellulose membranes (Amersham Biosciences Inc., St. Albans, United Kingdom), and then incubated with the primary antibody overnight. Subsequently, the membrane was incubated with anti-rabbit antibody conjugated with horseradish peroxidase (NA934V, Amersham Biosciences Inc.) and developed using the Amersham ECL system. Cell lysates of the Jurkat cells were used as the positive controls for Bim and Noxa [22]. In a prior study, we demonstrated that there is expression of Puma in the HCC2429 cell line [23].

### Statistical Analysis

The association between Bim, Noxa, or Puma and the categorical variables were analyzed by chi-square tests or Fisher's exact test, as appropriate. When assessing the effects of more than one factor on Bim or Noxa expression, we performed a multivariate logistic regression analysis. Survival curves were estimated using the Kaplan-Meier method, with differences in the survival distributions then evaluated using the log-rank test. The level of significance was set at p < 0.05. Statistical analyses were done using StatView 5.0.1 (SAS Institute Inc., Cary, NC, USA).

## Results

### Expression of Bim, Noxa, and Puma

**Figure 1:**
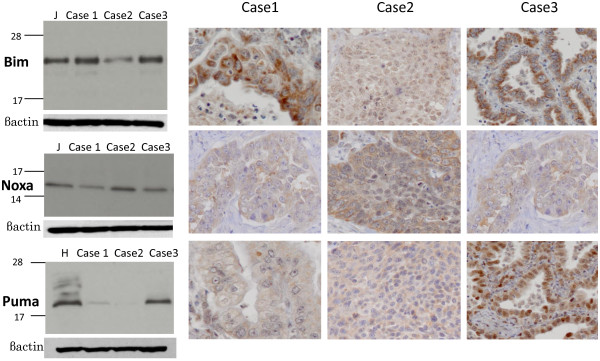
** Western blot analysis and immunohistochemistry of the surgical specimens obtained from three patients in the current cohort.** In the Western blot analysis, Bim was strongly detected in Cases 1 and 3, while Noxa was moderately found in Case 2, and Puma was strongly detected in Case 3. These findings match the results of the immunohistochemical staining analyses. Cell lysates of the Jurkat cells (J) were used as positive controls for Bim and Noxa. HCC2429 (H) was used as the positive control for Puma.

**Figure 2:**
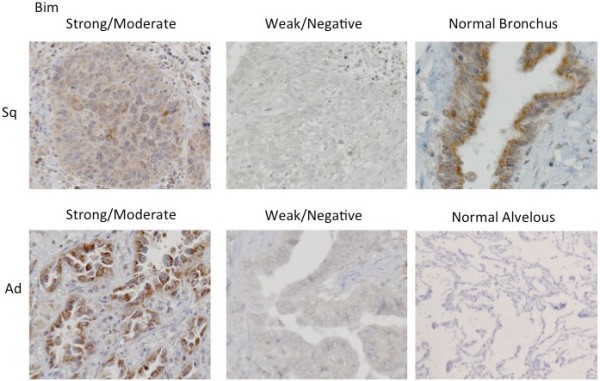
** Bim immunohistochemical staining pattern.** The immunohistochemical pattern for Bim in both the squamous cell carcinoma (Sq) and adenocarcinoma (Ad) samples.

**Figure 3:**
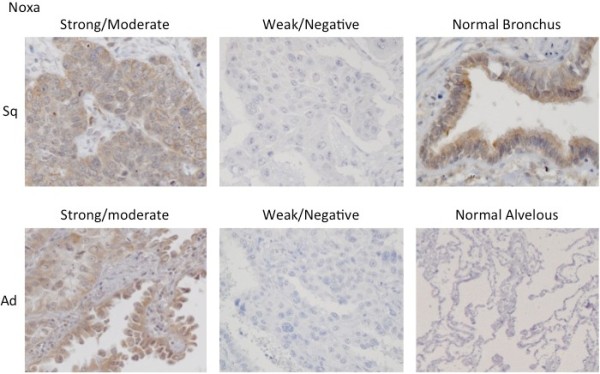
** Noxa immunohistochemical staining pattern.** The immunohistochemical pattern for Noxa in both the squamous cell carcinoma (Sq) and adenocarcinoma (Ad) samples.

**Figure 4:**
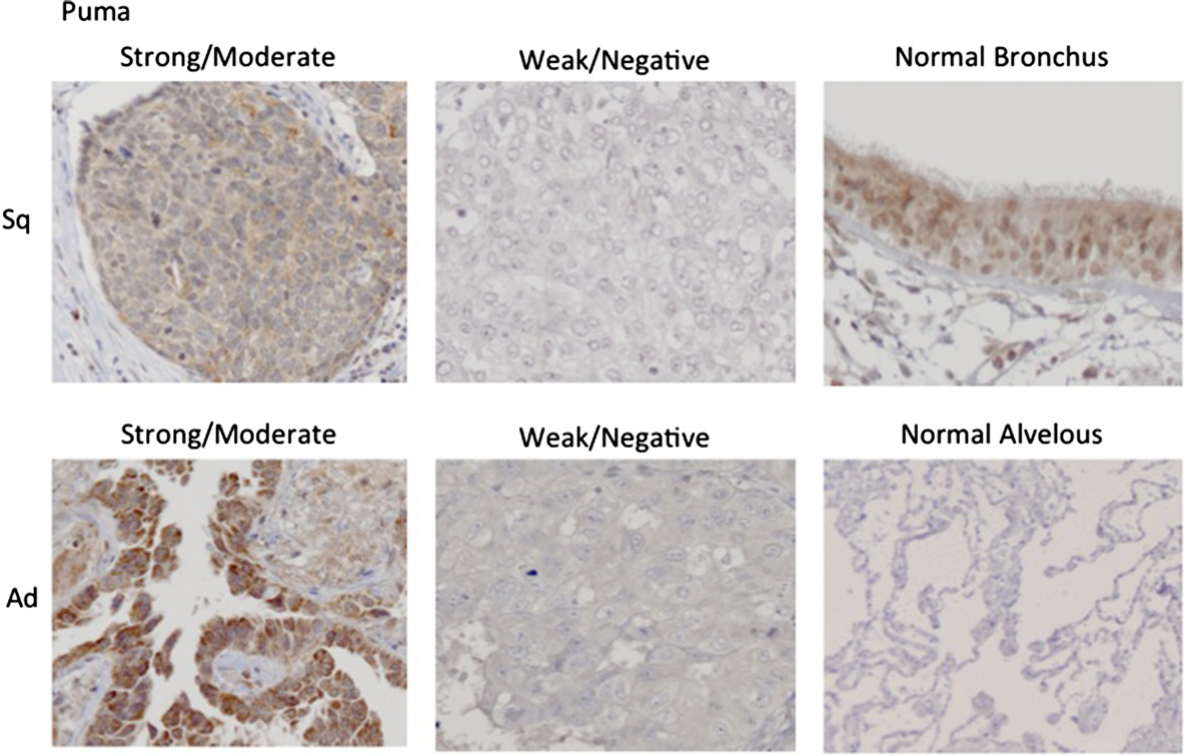
**Puma immunohistochemical staining pattern.** The immunohistochemical pattern for Puma in both the squamous cell carcinoma (Sq) and adenocarcinoma (Ad) samples.

The quality of the antibodies that were used in this study was first tested by Western blot analysis and immunohistochemistry using diverse cell lines and surgical specimens. Cell lysates of Jurkat cells were used as positive controls for Bim and Noxa [22]. In addition, we previously showed that there is expression of Puma in the NSCLC cell line, HCC2429 [23]. The expression of Bim, Noxa and Puma noted during the Western blot and immunohistochemistry analyses is seen in Figure 1. In three surgical specimens that were obtained from the study cohort of NSCLC, Bim was strongly detected in Cases 1 and 3, while Noxa was moderately found in Case 2, and Puma was strongly detected in Case 3. These data were consistent with the results of our immunohistochemical staining, with the staining for all of the antibodies observed within the cytoplasm of the cells (Figures 2,3 and 4). Bim showed homogenous staining within tumors. Noxa and Puma showed focal and heterogeneous staining within tumors. Normal bronchial epithelial cells, which were used as the internal positive control, showed moderate to strong expression of all BH3-only proteins. No expression of these proteins was noted in the normal alveolar pneumocytes.

### Correlation between the expression of the BH3-only proteins and the clinicopathological variables

We evaluated the relationship between Bim, Noxa, or Puma expression and the clinicopathological variables. High Bim expression was observed in 58 patients with NSCLC (43 %). This expression was shown to be associated with sex (p < 0.001), smoking habit (p = 0.03), pathological histology (p = 0.001), pathological T stage (p = 0.03), pathological disease stage (p = 0.02), and differentiation of tumor (Table 1). High Noxa expression was detected in 47 NSCLCs (35 %), with the expression linked to both smoking habit (p = 0.02) and pathological histology (p = 0.03) (Table 2). Although a high Puma expression was seen in 59 patients with NSCLC (44 %), this expression had no influence on any of the variables (Table 3). Multivariate logistic regression analysis used to examine the correlation between Bim expression and the clinicopathological variables demonstrated significant associations between both the low Bim expression and Sq histology (p = 0.04) and the high Bim expression with well differentiated tumors (p = 0.02) (Table 4). No link was observed between Noxa expression and either smoking habit or pathological histology in the multivariate logistic regression analysis (Table 5). When we further analyzed the relationship between BH3-only proteins and patient survival, we found that the expression of these BH3-only proteins was not correlated with the survival outcomes of any of the subgroups analyzed, including both the disease stage and the histological type.

**Table 1:** Correlation between Bim expression and the clinicopathological characteristics of 135 resected Non-small cell lung cancers

	**No. of Patients**		
**Characteristics**	**low**	**high**	**p**
Age			
<65	38	27	0.74
≥65	39	31	
Sex			
Men	61	30	<0.001
Women	16	28	
Smoker			
Never	16	22	0.03
Smoker	61	36	
Histology			
Adeno	32	42	0.001
Squamous	39	15	
Other1	6	1	
pT2			
T1	16	22	0.03
T2-4	61	36	
pN3			
N0	47	44	0.09
N1-3	30	14	
pStage4			
I	41	42	0.02
II-IV	36	16	
Differentiation			
Moderate/Poor	64	29	<0.001
Well	13	29	

^1^Other: adenosquamous cell carcinoma and large cell carcinoma.

^2^pT: pathological tumor classifications.

^3^pN: pathological nodal classifications.

^4^pStage: pathological disease stage.

**Table 2:** Correlation between Noxa expression and the clinicopathological characteristics of 135 resected Non-small cell lung cancers

	**No. of Patients**		
**Characteristics**	**low**	**high**	**p**
Age			
<65	39	26	0.22
≥65	49	21	
Sex			
Men	63	28	0.16
Women	25	19	
Smoker			
Never	19	19	0.02
Smoker	69	28	
Histology			
Adeno	43	31	0.03
Squamous	42	12	
Other1	3	4	
pT2			
T1	22	16	0.28
T2-4	66	31	
pN3			
N0	61	30	0.46
N1-3	27	17	
pStage4			
I	55	28	0.74
II-IV	33	19	
Differentiation			
Moderate/Poor	64	29	0.19
Well	24	18	

^1^Other: adenosquamous cell carcinoma and large cell carcinoma.

^2^pT: pathological tumor classifications.

^3^pN: pathological nodal classifications.

^4^pStage: pathological disease stage.

**Table 3:** Correlation between Puma expression and the clinicopathologic characteristics of 135 resectedNon-small cell lung cancers

	**No. of Patients**		
**Characteristics**	**low**	**high**	**p**
Age			
<65	34	31	0.81
≥65	42	28	
Sex			
Men	51	40	0.93
Women	25	19	
Smoker			
Never	21	17	0.88
Smoker	55	42	
Histology			
Adeno	45	29	0.06
Squamous	30	24	
Other1	1	6	
pT2			
T1	23	15	0.50
T2-4	53	44	
pN3			
N0	49	42	0.47
N1-3	27	17	
pStage4			
I	42	41	0.09
II-IV	34	18	
Differentiation			
Moderate/Poor	53	40	0.81
Well	23	19	

^1^Other: adenosquamous cell carcinoma and large cell carcinoma.

^2^pT: pathological tumor classifications.

^3^pN: pathological nodal classifications.

^4^pStage: pathological disease stage.

**Table 4:** Multivariate Logistic Regression Analysis for the correlation between Bim expression and clinical and clinicopathological characteristics

**Variable**	Adjusted OR* (95%CI**)	**P**
Sex (Men vs Women)	0.32 (0.09-1.17)	0.08
Smoker (Smoker vs Never1)	0.46 (0.30-1.80)	0.50
Histology (Sq2 vs Non-Sq3)	0.37 (0.17-0.84)	0.04
pT4 (pT2-4 vs pT1)	0.77 (0.34-1.97)	0.65
pStage5 (II-IV vs I)	0.47 (0.33-1.83)	0.12
Differentiation (Well vs Moderate/Poor)	5.12 (1.16-6.92)	0.02

*OR: odds ratio, **95%CI: 95% confidence interval.

^1^Never: never smoker.

^2^Sq: squamous cell carcinoma.

^3^Non-Sq: Included adenocarcinoma, adenosquamous and large cell carcinoma.

^4^pT: pathologic tumor classification.

^5^pStage: pathologic disease stage.

**Table 5:** Multivariate Logistic Regression Analysis for the correlation between Noxa expression and clinical and clinicopathological characteristics

**Variable**	Adjusted OR* (95%CI**)	**P**
Smoker (Smoker vs Never1)	0.49 (0.21-1.11)	0.09
Histology (Sq2 vs Non-Sq3)	0.63 (0.28-1.38)	0.25

*OR: odds ratio, **95%CI: 95% confidence interval.

^1^Never: never smoker.

^2^Sq: Squamous cell carcinoma.

^3^Non-Sq: Included adenocarcinoma, adenosquamous and large cell carcinoma.

### Correlation between BH3-only proteins and cellular biological expression

Results of previous studies on the expression of p27, Ki67 and cyclin E have implied that there is a prognostic significance for these expressions [24,25]. Therefore, using the same study cohort, we tested the correlation between the BH3-only proteins and these cellular biological expressions. Our results showed that a high Ki67 expression level was associated with a low Bim expression (p < 0.01), whereas there was no correlation between either the Puma or Noxa expression and any of the tested cellular biological expressions (Table 6). We also examined the correlation between the expression of Bcl-2 protein and BH3-only proteins, because the viability of cells is determined by the balance of expression of pro-apoptotic proteins such as BH3-only proteins and the expression of anti-apoptotic Bcl-2 family members,. However, there was no statistical correlation between Bcl-2 expression and BH3-only proteins. It suggests that other anti-apoptotic Bcl-2 proteins/BH3-only proteins axis may be involved in this study. Moreover, other survival pathway like MAPK or AKT/PI3K pathway may contribute to the cell viability.

**Table 6:** Correlation between Bim, Noxa or Puma expression and cellular biological expression

	**Bim**			**Noxa**			**Puma**		
	**low**	**high**	**p**	**low**	**high**	**p**	**low**	**high**	**p**
	**No. of Patients**			**No. of Patients**			**No. of Patients**		
Ki67									
High	56	28	<0.01	59	25	0.11	51	33	0.18
Low	21	30		29	22		25	26	
p27									
High	71	50	0.52	79	42	0.95	68	53	0.95
Low	6	8		9	5		8	6	
CyclinE									
High	45	29	0.33	49	25	0.78	45	29	0.24
Low	32	29		39	22		31	30	
Bcl-2									
High	32	25	0.97	37	20	0.95	37	20	0.51
Low	45	33		51	27		39	39	

## Discussion

In the current study, we examined the expressions of the BH3-only proteins, Bim, Noxa and Puma, and found that there was a correlation between these expressions and the clinicopathological features. While we also found that low Bim expression was significantly more prevalent in the Sq histology versus non-Sq histologu, this study revealed no significant correlations between any of the clinical variables and the expression of Noxa or Puma. Although there has been one pilot study that used immunohistochemistry to examine the expression of the BH3-only proteins, Bad, Bid and Bim, in NSCLC samples [17], the current study is the first to clarify the frequency of Bim, Noxa and Puma in a large series of NSCLCs.

In another study of NSCLCs that reported a similar pattern, the results showed that while a loss of Bim was observed in 2 out of 22 samples in the Sq histology and in 0 out of 19 in the Ad histology, the loss was not statistically significant [17]. Although many promising treatments have been recently developed for adenocarcinoma of the lung, including EGFR-TKI (tyrosine kinase inhibitor) and pemetrexed [1,26], there are few optimal treatments that can be used for Sq. However, our results suggest the possibility that the loss of Bim expression in Sq might be related to resistance to these anticancer drugs.

Bim has previously been shown to have a role in tumor suppression [12,13]. This is supported by our current data that showed a significant association between the loss of Bim expression and a high Ki67 expression, which is a marker of tumor proliferation. In addition, high Bim expression was correlated with well differentiated tumors. Taken together, this suggests that Bim may have an impact on both cell proliferation activity and tumor feature in NSCLC.

Bim has also been shown to be required for initiation of the apoptosis induced by EGFR-TKI in NSCLC cell lines with EGFR-TKI-sensitive mutations [27-29]. Furthermore, inhibition of Bim induction has also been observed in NSCLC with the EGFR-TKI resistance mutation, T790M, which suggests that Bim induction leads to the active response of the EGFR-TKI treatment [28]. Recently, BH3-mimetics have been developed and shown to have cytotoxic effects on many solid tumors [30,31]. The BH3-mimetic, ABT-737, along with its oral analogue, AB7-263, have especially been shown to promote apoptosis and suppress tumor growth in small-cell lung cancer [32]. Although it has been observed that several NSCLC cell lines show relative resistance to ABT-737 monotherapy [33,34], ABT-737 significantly enhanced EGFR-TKI-induced cell killing of NSCLC, and thus, this combined treatment might be a promising new therapy [29]. Although we have not examined the correlation between Bim expression and responses to treatment, our current data suggest that Bim expression might be involved with tumor proliferation and feature, which supports the notion that Bim expression could be a useful therapeutic marker.

Noxa and Puma expression levels have been investigated in various cancers, including colon cancer and ameloblastic tumors, and were shown not to have any clinical significance [35-37]. This is consistent with our current data. To the best of our knowledge, there has been little data reported concerning the relationship between these two BH3-only proteins and the various clinicopathological factors in NSCLC. Unlike Bim expression, these two proteins may have not been implicated in the pathogenesis of NSCLC, which suggests that further investigations of their roles are warranted.

Our findings also did not find any impact of the examined BH3-only proteins on survival outcomes. Likewise, there have been no other reports on any relationships between these proteins and patient prognosis in NSCLC. However, it has been verified that both Bim and Puma expression are independent prognostic factors in colon cancer [14]. Conversely, Bim expression has not been previously linked with patient prognosis in either gastric cancer or malignant mesothelioma [15,38]. Therefore, the origin and stage (early or advanced) of the tumor in addition to the subsequent therapies that have been used against the disease might account for the prognosis discrepancies that have been described. Moreover, these proteins are known to be regulated by many different complex upstream pathways, such as AKT/PI3K, MAPK, or p53 [8-11]. Thus, the various levels of pathway regulation that could potentially exist might also be contributing factors for the differences noted in the prognosis between the different types of malignant diseases.

## Conclusions

The current findings demonstrated that the loss of Bim expression was associated with both Sq histology and tumor aggressiveness in NSCLC.

## Authors’ contributions

JSK was involved in the design of the study, collected the clinical data, performed the immunohistochemical analysis and drafted the manuscript. SO co-drafted the manuscript. JK, EK and HM collected the clinical data. IK provided general support and helped to draft the manuscript. HDA provided general support and helped to draft the manuscript. MH supervised the study. All authors read and approved the final manuscript.

## Competing interests

The authors declare that they have no competing interests.

## Pre-publication history

The pre-publication history for this paper can be accessed here:

http://www.biomedcentral.com/1471-2407/12/286/prepub
